# Competence development as a means of HR management in the field of social work

**DOI:** 10.3389/fpsyg.2023.1212131

**Published:** 2023-07-24

**Authors:** Gabriela Ježková Petrů, Kristýna Zychová

**Affiliations:** Department of Management, Faculty of Economics and Management, Czech University of Life Sciences Prague, Prague, Czechia

**Keywords:** competency model, staff education, management education, professional competence, social services, social work

## Abstract

Professional competences in social services are becoming the subject of interdisciplinary cooperation and require a multidisciplinary approach. The research aimed to identify which professional competences are required by social service managers, to determine which variables influence the expansion of social work competences and to categorize the professional competences. Expansion of social work competences is influenced by many factors according to specific characteristics of the organization. The results were obtained through a questionnaire survey of managers (*n* = 247) and employees (*n* = 219). All organizations providing social services in the Czech Republic, according to the Register of Social Service Providers of the Ministry of the Interior of the Czech Republic, were contacted. The results were used for formulating a competency model for social services. The relationship between the selected variables and the expansion of social work competences was tested using a dependency test (*χ*^2^). The significant level of value was chosen as *α* = 0.05. The strength of dependence was calculated using a Cramer’s *V* measurement within 0 ≤ V ≤ 1. The result of the measurement was to test the validity of the hypothesis (hypothesis testing), and the result of the test was related back to the theory (verification). Factor analysis enabled the relationships between the latent variables to be uncovered and allowed two groups to be formed within the professional competences. Results showed a relationship between the expansion of competences through continuing education, the **evaluation of personal development** and **motivation plans**, and the **establishment of a set of professional competences** for each job. The results suggest that achieving a particular competency should be part of a workforce development plan. Factor analysis revealed two groups of professional competences. The first group includes competences such as socio-legal counseling, analytical, methodological, and conceptual activities in the social field, and social counseling and social rehabilitation. The second group includes competences such as professional activities in facilities providing social prevention services, social diagnostics, and identifying the needs of the population and the region.

## Introduction

Social work in human resource management is characterized by its use of advanced forms of working with people in the organization, i.e., soft models of human resource management (Li et al., 2015; Sojka, 2016; Armstrong and Taylor, 2020; Zhou and Guo, 2020). The essence of these models, which are based on the congruence of strategic management and HR strategy, is based on the participation and commitment of organizational members in managing the organization according to competences (Jongen et al., 2018). As the main elements of organizational development, people play a major role in their organizational activities. The growth and development of organizations today depend on the proper application of competences (Pourrashidi et al., 2017). Employees with their competences are considered an important tool for organizational change and development and are major factors in the success and failure of organizations (Yun, 2017). However, competences are rarely assessed comprehensively for a particular area. The issue of social workers’ competences is becoming more and more topical and is linked to the field of continuing education of social workers, which is a means of continuously acquiring, supplementing, and consolidating these competences. Competences become one of the key building blocks for a company’s success in achieving its mission, vision, and value creation (Sharma and Malodia, 2022). Social work is becoming one of the key areas of society. Social work has recently experienced a significant upsurge, especially during the COVID-19 pandemic, and the demands on the competences of social workers are related to this. This change in the inclusion of individuals in the organization’s management and the entire socioeconomic complex of the organization is based on the assumption of required education and the determination of qualifications and categorization of competences (Li et al., 2015; Urbanaviciute et al., 2020). Competences specify much better and, more precisely, the requirements not only for the sub-needs of continuing education (Trnka et al., 2020; Hajj et al., 2023) but also for the performance of the job. Thus, continuing education must lead to expanding such competences that can address a specific problem comprehensively in social work (Dwyer et al., 2015). The continuing education program should be set up by managers so that the acquired competences contribute to the ability to make the right decision based on sufficient information from relevant fields (Echegaray and García Martín, 2020). In real-world settings, continuing education programs often do not correspond with practice (Mousa and Othman, 2020), so it is important to address this area promptly. In the field of social work, managers should be required to have competences relevant to society’s current needs. Faber et al. (2012) point out a need for a more holistic and multidisciplinary focus on the issues. The notion of competence inconsistently appears in the literature. It is often confused with the concept of qualification (Martín et al., 2019; Echegaray and García Martín, 2020; Monteiro et al., 2020), which in the field of social work is based on statutory requirements for social work competences. A qualification is a capacity for a particular professional application and consists of individual competences (Matoušek, 2021). It comprises the knowledge, skills and competences that are necessary to perform the relevant activity. In social workers’ case, it is a combination of “core skills” and “thinking skills.” In the Czech environment, “core skills” are determined by the Act 108/2006 Coll. on Social Services. Competences are acquired by the individual in a lifelong learning process and are linked to the process of socialization. The worker acquires new competences during his/her working life, although he/she may not use them for the job. Core competences, “key qualifications,” can be characterized as core qualifications and “core skills” (Baizak et al., 2017). Competences are a combination of knowledge and skills appropriate to a particular context, whereas core competences are those everyone needs for personal development, social inclusion, and working life (Parks et al., 2020). In the context of the changing requirements for the performance of social services, it is necessary to devote sufficient space to the topic of competences and to define competences in the professional fields. Professional competences are based on “key qualifications” and are acquired during the social worker’s studies and continuing education. On the other hand social skills can be characterized as “thinking skills,” which are referred to as “functional skills” (Vasconcelos, 2020), see professional competences in Table 1.

**Table 1:** Professional competences for social work.

Professional competences
Social counseling and social rehabilitation
Socio-legal counseling
Coordination of the provision of social services
Analytical, methodological, and conceptual activities in the social field
Solving socio-legal problems in social care institutions
Social diagnostics
Providing crisis assistance
Professional activities in facilities providing social prevention services
Screening activity
Identifying the needs of the inhabitants of the municipality and the region

Source: Own elaboration (2023) based on Act 108/2006 Sb. on Social Services.

The competences can be characterized in three areas relevant to the presented research and complemented by examples from the social services on which the research is focused. Competence is always multidimensional, comprising a diverse mix of abilities, skills, and information (Andreev, 2018). In the case of social services, it reflects their multidisciplinary concept, where information from the fields of social, psychological, legal, crisis assistance, etc., is gathered, and the importance of a correct assessment of the situation is very crucial. Competence is defined by a standard, where the expected level of mastery of the competency is given in advance, with a set of performance criteria defined in advance, allowing the individual to demonstrate and be able to evaluate their competence (Takawira, 2020). In social services, we could demonstrate this aspect by performing certain activities—e.g., crisis intervention and legal counseling—which fall within professional competences and can only be performed by a worker if he or she has expert knowledge in these areas.

Each competence has the potential for action and development, i.e., each competency is acquired and developed through the processes of education and long life learning (Siegelman et al., 2017). For example, in social services, competences are acquired in tertiary education. Some are then supplemented or fostered through continuing education. This concept implies that each competence is a unique characteristic of a person enabling him to act successfully and further develop his potential. Job competences are essential for the performance of the job and are examined in the area of professional competences. Competences must be viewed in a multidisciplinary way. A social worker should also have social competences. Competences can also be conceptualized multidimensionally, as they comprise a diverse mix of abilities (Rogat and Linnenbrink-Garcia, 2019; Brown et al., 2020). In social work, professional competences are defined within the framework of Act 108/2006 Sb. on Social Services. The required education defines a certain standard, where mastery of the competences is predetermined. In the case of social workers, by completing a relevant statutory training program. Where competence is characterized as a potential for action and development, each competence is acquired and developed in education and learning processes. In this respect, in the field of social work, the means for education and learning is continuing education, which is provided for and characterized by law for this field.

Continuing education can be defined as a process that aims to increase the competences and attitudes of employees toward work and to increase the organization’s effectiveness (Koskimäki et al., 2020). Competences are the basic building blocks of a talent management and workforce training system. Each employee exhibits certain behaviors, skills, knowledge, and other expectations critical to the success of the employee and the organization (Rogat and Linnenbrink-Garcia, 2019). Competences are observable phenomena through which an employee achieves the desired performance (Stadick, 2020) and must be consistent with ethical behavior (Vasconcelos, 2020). Competences can be gained, developed, or lost. They also depend on the employee’s age and the cultural environment in which the person works. Bogren et al. (2020) state in their study that higher education programs, both undergraduate and graduate, should be designed to meet a given international standard within an international context. These programs should be designed to allow both undergraduate and graduate students to achieve the highest possible level of competences that can be built upon and further enhanced. Competences should address the learning objective, reflect expectations that match the external image of the educational program, and should be expressible in terms of measurable behavior. A standard independent of other learners’ performance should be used to assess competence. Workers should be informed about what is expected of them (Baizak et al., 2017; Rogat and Linnenbrink-Garcia, 2019; Vasconcelos, 2020).

At a time when employability issues are the cornerstone of society, job competences are the most important factor in satisfaction with the educational process. The question of how universities and continuing education courses can better prepare graduates for the labor market is thus becoming increasingly crucial (Baizak et al., 2017). Furthermore, it is important to consolidate acquired competences in practice, i.e., universities should support the acquisition of competences by integrating students into training and practice (Monteiro et al., 2020). Matthews (2020) further states that these programs should be designed to allow both undergraduate and postgraduate students to achieve the highest level of competence that can be built upon in practice and further enhanced.

A predetermined structure in which the competency development is embedded and corresponds to the profession and practice is crucial for subsequent competency development (Bogren et al., 2020). Fullerton et al. (2013) point to the requirement for a single definition and identity for the profession that should be shared within the professional community. Within this professional community, a set of competences that professions should possess should be shared and generally accepted (Fullerton et al., 2013). This requirement for a single definition and identity within the professional social work community is partially fulfilled through the activities of the Professional Chamber of Social Workers of the Czech Republic. However, the results of the research should help to identify individual competences more precisely.

The competency model cannot cover the entire content of the work activity, but it is an important part of it. It is used precisely to determine workers’ development plans or goals. Its further use lies in the form of a tool for controlling the educational process, the direction of educational management, and the process of personnel management. The competency model assessment aims to match the assessor and the assessed in the following areas:

- Knowledge of the current level of competence;
- Required level of competence;
- Setting employee development goals and plans;
- A method of cooperation between the supervisor and the worker. These four areas were chosen as the basis for research on competences concerning continuing education in social services.

In daily work, it is necessary to work with emotional stress, work behavior, and work performance, which affect the consolidation of competences. Therefore, social competences are a very important part of the professional competences they complement (Solé Blanch, 2020). Within the framework of consolidating and expanding social and professional competences in the field of social work, adequate reactions within social ties are created and acquired, i.e., within communication in society, in a team, with clients and other persons, e.g., from public administration, sponsors, etc. (Viens et al., 2020; Zhou and Guo, 2020).

The article’s main aim is to propose a model of competence development for social workers. The sub-objectives are to identify the factors that influence the development of competences in social work organizations, to categorize the professional competences and to identify which professional competences are required by social service managers.

*RQ1: Which professional competences are required by social service managers?*

*RQ2: What factors influence the expansion of competences?*

*RQ3: Is it possible to categorize or identify groups of professional competences?*

## Materials and methods

### Research methods and measurement validation

The quantitative research used structured data collection in the form of questionnaires. A total of two questionnaire surveys were conducted. The first one focused on managers in social services. The second focused on ordinary social workers. This type of research is associated with the hypothetical-deductive model, where a formal statement is first made to explain relationships in the real world. These relationships were formulated based on the results of qualitative research and theoretical findings. Then deduction was made, and if the theory was valid, a relationship could be found between at least two variables X and Y (hypothesis) (Bacon-Shone, 2013). The results were formulated into tables to clarify the interrelationships between variables and competences. Relationships between variables were measured using statistical tools—the test of dependence (*χ*^2^) and Cramer’s *V* measurements. The significant level of value was chosen as *α* = 0.05. The strength of dependence was calculated using a Cramer’s *V* measurement within 0 ≤ V ≤ 1. The result of the measurement was to test the validity of the hypothesis (hypothesis testing), and the result of the test is related back to the theory (verification). Finally, a set of variables was identified for the domain based on the research, where the relationship between the variable and competence enhancement was examined. The variables were: the sector in which the organization operates; whether it is part of a larger group of organizations or operates independently; the size of the market in which it operates; and the size of the organization by the number of employees. The classification of enterprise size was according to the European Commission Recommendation (No. 2003/361/ES). Based on the specific nature of the Czech education environment in social services and the activities of organizations in the social field, basic variables that may influence the expansion of competences in the social field were selected. The nature of the social work sector of activity of the organization, separate organization/network of organizations, size by the number of employees and the market in which it operates influence the education system in social services from the long-term perspective (Matoušek, 2021). Specific variables were identified through a qualitative study using a case study conducted in a social service organization. Based on this study, the set of specific variables used in our research was identified. In addition, specific variables that were identified as significant within the field of social work were measured to determine whether they influence the expansion of competences (see basic and specific variables in Table 2).

**Table 2:** Set of investigated variables.

Basic variables	Specific variables
Sector of activity of the organization	Participation in further education
Separate organization/network of organizations	Motivation from the organization’s leaders
Size by number of employees	Evaluating continuing education
The market in which it operates	Personal development plans
	Existence of an HR department
	Use of digital technologies
	Integrating continuing education into the long-term strategy of the organization
	Evaluation of staff development and incentive plans
	Evaluation of further education objectives
	Establishing the competency framework for the position

Source: Own elaboration (2023).

The results of the measurements were grouped into tables to show Cramer’s *V* and the strength of the dependence according to de Vaus (2014), as well as the result of the rejection of the hypothesis. We ensured that the measurements were valid and reliable. The data was cleaned, converted into an Excel spreadsheet, and processed using the statistical program IBM SPSS 26. The results obtained by descriptive statistics were followed by factor analysis to identify the relationships between variables. Factor analysis allows the variance of observed variables to be explained by fewer latent variables. Factor analysis has been used, although it may present potential problems arising from its shortcomings, such as the risk of spurious results in the routine use of factor analysis, ambiguity in the solution or ambiguity in the estimation of factor parameters, excessive subjectivity, and unclear interpretation and approximation of results. As an advantage, quantitative research is straightforward data collection that is accurate and numerical. In the case of contacting organizations in social services, all organizations in this area were contacted through the Register of Social Service Providers in the Czech Republic. The total number of questionnaires in the survey among social service managers was 1,168, of which the return rate was 247 questionnaires, i.e., 21%. As part of the survey of social worker employees, 965 questionnaires were sent out, of which the return rate was 219, or 23%. The disadvantage and limitations of the questionnaire survey are that the theories used by the researcher may not correspond to the local practices, and the researcher may omit phenomena because he/she focuses only on a particular theory.

Last, the acquired knowledge may be too general for application in local conditions (Hendl, 2005). The fact that the return rate of the questionnaires was lower than expected in the research may also be a limit to the conclusions. The questionnaire was built using the Survio platform, and a link to the questionnaire was included in the accompanying email.

### Research sample

A total of 1,168 questionnaires were sent out via email to the organizations’ senior managers or executive management, asking them to for completion. As a result, 247 questionnaires were returned, i.e., 21% (Table 3).

**Table 3:** Basic data about social service managers.

Characteristics	Category			
Sector	Public		Church	Private
	188 (76.1%)		39 (15.8%)	20 (8.17%)
Market	Regional	Local	National	Global
	143 (57.9%)	63 (25.5%)	37 (15%)	4 (1.6%)
Part of a larger group of organizations	Yes		No	
	120 (48.6%)		127 (51.4%)	
Organization size (% of employees)	1–9	10–49	50–249	250 and more
	59 (23.9%)	90 (36.4%)	91 (36.8%)	7 (2.8%)
Existence of an HR department	Yes		No	
	53 (21.5%)		194 (78.5%)	

Source: Own elaboration (2023).

The data were obtained through quantitative research, which was conducted using a questionnaire survey method (*n* = 219). A total of 965 questionnaires were emailed to the organizations’ managers or executive management, asking social workers to complete the questionnaire. Two hundred and nineteen questionnaires were returned, i.e., 23% (Table 4).

**Table 4:** Basic data about social workers.

Characteristics	Category			
Sector	Public		Church	Private
	154 (70.3%)		37 (16.9%)	28 (12.8%)
Market	Regional	Local	National	Global
	125 (57.1%)	43 (19.6%)	38 (17.4%)	13 (5.9%)
Part of a larger group of organizations	Yes		No	
	114 (52.1%)		105 (47.9%)	
Organization size (% of employees)	1–9	10–49	50–249	250 and more
	28 (12.8%)	87 (39.7%)	81 (37.0%)	23 (10.5%)
Existence of an HR department	Yes		No	
	184 (84.0%)		35 (15.98%)	

Source: Own elaboration (2023).

## Results

**The first research question** focused on professional competences and explored which professional competences managers most demand from their employees. The research set of professional competences for social workers was taken from the statutory competences of social workers.

As shown in Table 5, most managers require employees to have competences in the field of social counseling and social rehabilitation in 145 cases (58.7%), followed by socio-legal counseling in 130 cases (52.6%), coordination of social services in 105 cases (42.5%) and analytical, methodological and conceptual activities in the social field in 103 cases (41.7%).

**Table 5:** Required professional competences by manager.

Professional competences	Total responses	%
Social counseling and social rehabilitation	145	58.7
Socio-legal counseling	130	52.6
Coordination of social services	105	42.5
Analytical, methodological, and conceptual activities in the social field	103	41.7
Solving socio-legal problems in social care institutions	93	37.7
Social diagnostics	85	34.4
Providing crisis assistance	85	34.4
Professional activities in facilities providing social prevention services	79	32
Screening activity	66	26.7
Identifying the needs of the inhabitants of the municipality and the region	53	21.5

Source: Own elaboration (2023).

From the point of view of social work managers, it is evident that there is a great demand for competences related to social counseling and socio-legal counseling, coordination of social service provision, and analytical, methodological, and conceptual activities.

**The second research question** aimed to establish the relationships between the expansion of professional competences among social workers and the variables sector of operation of the organization, whether the organization is part of a larger entity, market of operation, and size of the organization. Four separate tests were conducted to answer the question, the results of which are shown in the table. In addition, the interrelationships between the variables could be explored by multivariate analysis, which would uncover additional relationships and could be the subject of further follow-up research.

In the first part of the research, the following hypotheses were tested:

- H_0_1: The development of professional competences through continuing education in an organization does not depend on the business sector.
- H_0_2: Expanding professional competences through continuing education in an organization does not depend on whether the organization is part of a larger group of organizations.
- H_0_3: Expanding professional competences through continuing education in the market in which the organization operates.
- H_0_4: The development of professional competences through continuing education in an organization does not depend on the size of the organization.

Subsequently, the relationships between the expansion of professional competences and these variables were evaluated, and the set was determined based on qualitative research (Tables 6, 7):

- Participation in continuing education;
- Motivation from the leaders of the organizations;
- Evaluation of continuing education;
- Personal development plans;
- The existence of an HR department;
- Use of digital technologies;
- Integrating continuing education into the long-term strategy of the organization;
- Evaluating the impact of continuing education in the organization;
- Evaluating goals and planning for further education;
- Establishing the competency framework for the position;
- Existence of Age Management.

**Table 6:** Dependency test results.

Characteristics	Sector	Part of a larger group	Market	Size
	*p*-value/Cramer’s *V*			
Expansion of professional competences	0.0374/−	0.541/−	0.805/−	0.731/−

Own elaboration (2023).

**Table 7:** Results of Person’s *Chi-square* test for the tested hypotheses H_0_1–H_0_4.

No.	There is no dependence between	Asymp. significance	Decision (rejection of H_0_)	Cramer’s *V* value	The power of dependence according to de Vaus
H_0_1	Expansion of professional competences through continuing education and the sector in which the organization operates	*p* = 0.374	No	X	X
H_0_2	Expansion of professional competences through continuing education and whether the organization is part of a larger group of organizations	*p* = 0.541	No	X	X
H_0_3	Expansion of professional competences through continuing education and the market in which the organization operates	*p* = 0.805	No	X	X
H_0_4	Expansion of professional competences through continuing education and the size of the organization according to the number of employees	*p* = 0.731	No	X	X

Source: Own elaboration (2023).

In the second part of the research, the following hypotheses were tested:

- H_0_5: The development of competences through continuing education in an organization does not depend on whether employees participate in continuing education.
- H_0_6: Expanding competences through continuing education in an organization does not depend on being motivated by a manager.
- H_0_7: The development of competences through continuing education in the organization does not depend on the evaluation of further education.
- H_0_8: The development of competences through continuing education in the organization does not depend on the employee’s personal development plan.
- H_0_9: Expanding competences through continuing education in an organization does not depend on the use of digital technologies.
- H_0_10: Expanding competences through continuing education in an organization does not depend on not integrating further education into the long-term strategy of the organization.
- H_0_11: The development of competences through continuing education in the organization does not depend on evaluating the impact of continuing education in the organization.
- H_0_12: The development of competences through continuing education in the organization does not depend on assessing the objectives of continuing education.
- H_0_13: The development of competences through continuing education in an organization does not depend on the existence of defined professional competences for a given position.
- H_0_14: The development of competences through continuing education in an organization does not depend on the existence of an HR department.

The results presented in Table 6 give the *p*-value of the dependence and a summary of the rejection of the formulated hypotheses.

Based on the results of the Pearson *Chi-square* test, it can be summarized that the expansion of professional competences through continuing education

- does not depend on the sector in which the organization operates;
- does not depend on being part of a larger organization;
- does not depend on the size of the organization according to the number of employees;
- does not depend on the market in which the organization operates.

Next, the relationships (Table 8) of competence extension and the variables were tested:

- participation in further education;
- motivation from the leaders of the organizations;
- evaluation of continuing education;
- personal development plans;
- existence of an HR department;
- use of digital technologies;
- integration of continuing education into the organization’s long-term strategy;
- evaluation of staff development plans and incentive plans;
- evaluation of further education objectives;
- establishment of the competency framework for the position.

**Table 8:** Results of Person’s *Chi-square* test for the tested hypotheses H_0_5–H_0_14.

No.	There is no dependence between	The *p*-value	Decision (rejection of H_0_)	Cramer’s *V* value	The power of dependence according to de Vaus
H_0_5	Expansion of professional competences through continuing education and participation in continuing education	0.691	No	X	X
H_0_6	Expansion of professional competences through continuing education by motivating staff through management	0.219	No	X	X
H_0_7	Expansion of professional competences through continuing education and evaluation of continuing education	0.339	No	X	X
H_0_8	Expansion of professional competences through continuing education and a staff development plan	0.809	No	X	X
H_0_9	Expansion of professional competences through continuing education and opportunities to use digital technologies	0.911	No	X	X
H_0_10	Expansion of professional competences through continuing education and integrating continuing education into the organization’s long-term strategy	0.318	No	X	X
H_0_11	Expansion of professional competences through continuing education and evaluation of staff development plans and incentive plans	0.019	Yes	0.151	Low to medium dependence
H_0_12	Expansion of professional competences through continuing education and assessing continuing education objectives	0.304	No	X	X
H_0_13	Expansion of professional competences through continuing education and establishing job-specific competences	0.004	Yes	0.188	Low to medium
H_0_14	Expansion of professional competences through continuing education and motivation	0.335	No	X	X

Source: Own elaboration (2023).

Table 8 summarizes the results of all the hypotheses examined and presents the results of Cramer’s *V* and the strength of the dependence.

- Expansion of professional competences through continuing education does not depend on participation in continuing education.
- Expansion of professional competences through continuing education does not depend on staff motivated by the organization’s leaders.
- Expansion of professional competences through continuing education does not depend on whether the organization evaluates continuing education.
- Expansion of professional competences through continuing education does not depend on having a development plan.
- Expansion of professional competences through continuing education does not depend on the worker’s use of digital technologies.
- Expansion of professional competences through continuing education does not depend on whether continuing education is included in the organization’s strategic plans.
- Expansion of professional competences through continuing education **depends** on whether staff development and incentive plans are evaluated (the strength of the dependency is 151 low to medium).
- Expansion of professional competences through continuing education does not depend on whether the objectives of further education are assessed.
- Expansion of professional competences through continuing education **depends** on whether the competences for the job are established (strength of dependence 0.188—low to medium).
- Expansion of professional competences through continuing education does not depend on whether there is an HR department in the organization.

Based on the dependency results (Table 8), the dependency between the expansion of professional competences through continuing education was shown to be dependent on whether staff development plans and incentive plans are evaluated and whether professional competences are set for the job. Confirming these dependencies is an important element for the management of continuing education.

**Research question 3** aimed to categorize the professional competences of social workers. Factor analysis of professional competences was used to answer this question.

Factor analysis revealed 2 factors (Table 9), the first of which explained the behavior of 28% of the social worker sample and the second of which explained the behavior of 15% of the sample.

**Table 9:** Results of factor analysis.

Factor	Total variance	Total % of variance	Cumulative % of variance
1	3.228	28.365	28.365
2	1.554	15.118	15.118

Source: Own elaboration (2023).

The first factor (Table 10) groups the variables (professional competences used) socio-legal counseling (0.804), analytical, methodological, and conceptual activities in the social field (0.776), and social counseling and social rehabilitation (0.688). The coefficient values for the first factor range from 0.804–0.688. This factor has been called “Socio-legal competence.” The second factor called “Social work competences” explains 15% of the sample of social workers and includes competences such as professional activities in social prevention services (0.803), social diagnostics (0.774), and identifying the needs of the inhabitants of the municipality and the region (0.643).

**Table 10:** Detailed results of the factor analysis.

Variable	Factor 1	Factor 2
Social diagnostics	0.095	0.774
Solving socio-legal problems in social care institutions	0.681	−0.165
Socio-legal counseling	0.804	0.072
Analytical, methodological, and conceptual activities in the social field	0.776	0.009
Professional activities in facilities providing social prevention services	0.170	0.803
Screening activity	0.643	0.136
Providing crisis assistance	0.558	0.217
Social counseling and social rehabilitation	0.688	0.192
Identifying the needs of the inhabitants of the municipality and the region	−0.234	0.643
Coordination of social services	0.484	0.240
**Total % of variance**	**28.365**	**15.118**
**Factor**	**Socio-legal competences**	**Competence in social services**

Source: Own elaboration (2023).

Statistically, the most significant factor is 1 called “Socio-legal competence,” which explains the behavior of 28% of the sample of social workers. In this case, the variables of socio-legal counseling, analytical, methodological, and conceptual activities in the social field, and social counseling and social rehabilitation are explained. These competences are characterized by a common element, namely socio-legal activities. Socio-legal activities are carried out within the framework of social counseling, but also in direct care facilities and are also part of screening activities. At the same time, its knowledge builds on the analytical, methodological, and conceptual activities that are an important part of social work. Basic social counseling is part of all types of social areas. At the same time, it also includes professional counseling, which is provided within the framework of civil counseling centers and marriage and family counseling centers. This includes counseling for victims of crime and domestic violence and legal advice for people with disabilities and the elderly. In addition, the service provides counseling and mediation of contact with the social environment, therapeutic assistance, and help promoting human rights. All services are provided within the framework of telephone crisis intervention, low threshold facilities for children and youth, crisis assistance, and activation services for families with children (Gavurova and Tarhanicova, 2021).

The second factor “Competence in social services” describes the behavior of 15% of the sample. This includes competences such as professional activities in facilities providing social prevention services, social diagnostics, and identifying the needs of the inhabitants of the municipality and the region. Social prevention services are designed to prevent social exclusion. The aim is to help the client overcome a socially unfavorable situation and to avoid the occurrence and spread of unwanted societal social phenomena. Social diagnosis focuses on the general recognition of social problems. It is a concept that helps to detect or recognize the spread of socially pathological phenomena in the community. The survey aims to identify the individual needs of municipalities and towns, considering the current problems and social situation in the area. This factor describes competences focused on social work, with a focus on screening and direct social work. In this context, it is important to define the objective of continuing education, its methods, and its evaluation in a way consistent with the objectives of the development plan. Al-Kurdi et al. (2020) refer to this approach as a set of modules leading to the achievement of competences.

The results of the dependency test confirmed that there is a dependency between the expansion of competences and whether staff development plans and motivation plans are evaluated, as well as on whether professional competences are set for the job. The results of the factor analysis revealed that there are two groups within the competences. One group consists of workers who focus on socio-legal competences, and the other group includes workers who focus on competences aimed at the direct performance of social work. The combination of these competences should lead to the fact that they should be taught in continuity, taken into account in development plans and the design of the incentive plan.

The area of professional competences is a key area in social services. From the overall perspective of continuing education, selecting the method, setting the continuing education objective, and evaluating continuing education should lead to achieving the required competences, maintaining existing competences, or self-development. Ensuring that competences are aligned with the current and future needs of the organization is a key aspect of learning development. At the same time, we need to recognize that we can predict future labor needs and skills through surveys or experience. The reality created by societal developments cannot be modeled (Krasna et al., 2020). This fact has been confirmed for us in the current context, where the social services sector has been hit by the COVID-19 pandemic or the influx of refugees from Ukraine, which had no previous example, and it was not possible to build on the best practice models and examples cited by Tomuletiu et al. (2009). It is, therefore, necessary to adopt a new paradigm of continuing education for the future.

From managers’ point of view, the most important competences are in the **social counseling and socio-legal counseling, coordination of the provision of social services, and analytical and conceptual activities**.

**Figure 1:**
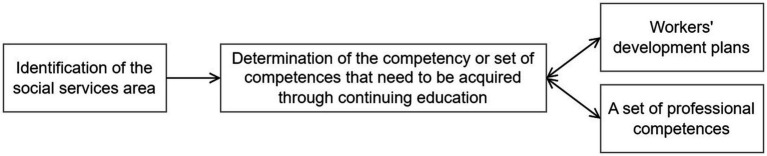
Competence in social services. Source: Own elaboration (2023).

The interrelationships examined between competence enhancement confirmed the relationship between competence expansion through continuing education, **staff development plans**’ evaluation, and the **establishment of job-specific competences**. These results suggest that managers should focus more on developing staff development plans and creating a set of competences that are appropriate for the social worker’s position, given the area in which they work. Based on the research results, it was possible to draw up a scheme for determining competences for social workers, shown in Figure 1.

The results confirmed that the determination of the set of competences to be complemented through continuing education is dependent on the social services sector. As illustrated in Figure 1, the first step should be to identify the area of social services that the worker performs. Based on this identification, the competences that need to be supplemented for the performance of a given position or job through continuing education would be determined. This addition would be based on a set of predefined professional competences and would also be in line with the worker’s development plan. This concept should be developed by the HR department for all areas of social work provided by the organization.

A predetermined structure is important for the systematic expansion and development of competences, which is confirmed by the requirement for a set of professional competences for the position. At the same time, this set needs to be consistent and accepted across the organization. For example, in the case of social service organizations, it could be part of continuing education management.

In light of the results obtained, the following competency recommendations can be proposed:

- The organization should develop a **set of required competences** for the job based on the area of social work on which the job is focused. This set of competences should be updated at regular intervals by the manager. The update and evaluation should then be an incentive to incorporate new professional competences into this set according to the development of the services provided, the requirements of the clients, and the inclusion of another perspective in the solution of the problem (i.e., ensuring a multidisciplinary approach). The set of professional competences should be appropriately complemented by social competences, which is based on the practice and experience of managers and is also the subject of continuing education.
- As part of the expansion and consolidation of competences, a **worker development plan** should be drawn up that reflects both the requirements in the set of professional competences and the requirements for self-development. In continuing education, the main objective is not only to increase and consolidate competences, but also self-development, as has been confirmed in research on the objectives of further education. The **evaluation of development plans** has a major impact on the expansion of competences, as confirmed by the quantitative research results. At the same time, it is a management tool that allows for flexibility and the right direction in employee development. The development plan can also be described as a **motivational tool** that, together with appropriate forms of continuing education, can reduce the turnover of social work staff.
- Continuing education in the area of professional competences is possible in two professional competences groups. The first group is represented by socio-legal counseling, analytical and conceptual activities, and social counseling and rehabilitation. The second group focuses mainly on social services activities, including professional activities in social work facilities, social diagnostics, and identifying the population’s needs and the region.

In the expansion of professional competences, there is a need to ensure the cooperation of the manager or HR officer and the employee in identifying the development area. The supervisor’s task would be to identify the worker’s talents, who would then receive continuing education in the field of social work or social work methods. The research results have shown a clear definition of these two areas in professional competences and, at the same time, have presented two paths that should be followed in expanding competences. The first path is characterized by socio-legal counseling and legal overview; the second by social activities such as social diagnosis and crisis intervention. The assessment of these areas and the subsequent selection of competences should correspond to the job and be incorporated into staff development plans.

## Discussion

In the area of social services, one can agree with Bogren et al. (2020), who stated that the regulated structure, or compliance with the legal standards in which the competency enhancement is embedded, which corresponds to the profession and practice, is crucial for subsequent competency enhancement (Bogren et al., 2020). The research confirmed that social work had established professional competences within the law and that expanding competences is one of the goals of continuing education. The conclusions reached in the research by Fullerton et al. (2013) can be applied to the findings in the social services field. Their study pointed to the need for a single definition and identity for the profession, which should be shared within the professional community. Within this professional community, a set of competences that professions should possess should be shared and generally accepted (Fullerton et al., 2013; Baizak et al., 2017). In the field of social work, there are so-called professional competences that a social worker must possess. These professional competences are listed in Act No. 108/2006 Sb., on Social Services. The results of the research show that there are two areas of professional competence, the socio-legal area and the area focused on the performance of social work, which enable the inclusion of individuals in the whole socio-economic system of the organization. These areas are based on the assumption of compulsory education and the determination of qualifications (Viens et al., 2020). The categorization of competences contributes to the correct setting of workers’ training plans and makes it easier to identify further training needs (Trnka et al., 2020). Professional competences should be combined so that the worker can address a specific social problem in a complex and multidisciplinary manner (Dwyer et al., 2015). In practice, the resulting categorization of professional competences can enable correct action and decision-making by a social worker who has to process a large amount of information from relevant disciplines (Echegaray and García Martín, 2020). In natural settings, the system of competence enhancement often does not correspond to practice (Mousa and Othman, 2020).

As Krasna et al. (2020) state, a key aspect of educational development is ensuring that competences align with current and future needs. At the same time, it is important to recognize that while we attempt to predict future workforce needs through surveys or historical data, the reality created by population change cannot be modeled from past trends, and therefore we must adopt new educational paradigms for the coming future (Krasna et al., 2020). In contrast to Bell (2010), Krasna et al. (2020) had not previously proposed significant changes to social work education but examined and evaluated well-established precedents and offered practical suggestions where a particular type of competency and associated skills could be naturally incorporated into existing elements of social work education and subsequent training. As Bell (2010) notes in his study, population impacts still need to be generally incorporated into social work education and training systems for social service workers. However, the necessary competences could be added to existing models and best practices in social work (Bell, 2010). The same theory is put forward by Tomuletiu et al. (2009), who base experience sharing on the so-called good practice model and refer to it as one of the most important models in professional learning (Tomuletiu et al., 2009). However, the practice of social work requires a worker with a range of professional competences based on specific personality traits. Research has shown, in agreement with the findings of Ivry et al. (2005), that the exact mix of required competences varies depending on the level of the position performed and the tasks addressed. Research among managers has clearly shown that workers rarely have the required competences, and organizations need to plan and implement continuing education to acquire the necessary competences. The research results showed the need to compile a set of professional competences for the individual activities of a social worker. Based on the results, it would be possible to create a combination of professional competences that would reflect the job’s specifics and correspond with practice (Mousa and Othman, 2020). These combinations of professional competences could be consolidated in the framework of the social worker development plans drawn up as part of continuing social work education. Our research also confirmed the need to evaluate social worker development plans and their linkage to the enhancement of professional competences. As society evolves, the changing structure of social services requires the consolidation and expansion of professional competences for their use to be of higher quality and effectiveness (Matthews, 2020). By incorporating the expansion and consolidation of competences into social worker development plans and evaluating them regularly, the management of organizations gains a unique tool to train social workers effectively.

Further research directions should move toward social worker development plans that reflect specific competency requirements as revealed by the dependency test and factor analysis. In addition, further research should be directed toward evaluating workers’ development and competency acquisition and evaluating training programs. Finally, further research should also move toward linking the disciplines taught within university education and within continuing social work education offered by accredited institutions.

## Conclusion

The aim of the research was to determinate professional competences in the field of social services. To compile a set of professional competences, we used Act No. 108/2006 Sb., on Social Services and did a further extensive literature search. Then, research questions were formulated for the competence area and were answered using descriptive statistics, dependency tests, and factor analysis. Research on competences has shown that leaders most demand competences in the area of **social counseling**, **socio-legal counseling**, **coordination of social service provision**, **and analytical and conceptual activities**.

Subsequently, the research aimed to determine the degree of dependence between the selected variables and expanding professional competence. The question was answered using a dependency test. Results showed a relationship between the expansion of competences through continuing education and **evaluating personal development, motivation plans**, and **establishing a set of professional competences** for each job. Results suggest that achieving a particular competency should be part of a workforce development plan. Furthermore, research has demonstrated that there should be a set of required professional competences that are required for a particular job according to the field of social work. Findings were then implemented in a flowchart (Figure 1), representing the individual steps for achieving the required competences in practice.

The last part of the research focused on categorizing professional competences. The results were processed using factor analysis, revealing two professional competences groups. The first group is represented by socio-legal counseling, analytical and conceptual activities, and social counseling and rehabilitation. The second group focuses mainly on social services activities, including professional activities in social work facilities, social diagnostics, and identifying the population’s needs and the region. The factor analysis revealed two groups of professional competences, the first pointing to competences needed for socio-legal positions and the second to competences for social services.

The proposed scheme (Figure 1) contributes in practical terms to complementing competences to such an extent that the social worker can perform the required activities. The proposed scheme (Figure 1) also contributes to evaluating the achievement of competences and allows it to be linked to the evaluation of staff development.

Research results confirmed that two factors influence the field of competences in social services. The first is the evaluation of staff development and motivation plans, and the second is the definition of a set of competences for a given position. The combination of professional competences is dependent on the position performed. Competences can be described as the observable activities through which an employee achieves the desired performance. For social workers, possessing a given competence is necessary to perform social services.

Research results have shown that expanding competences in social services is subject to determining the set of professional competences required for a given job. Determining a set of competences for a given job, based on the area of social work that the worker performs, is a unique basis for acquiring core competences. Even in social work and social service provision, competence is an organization’s competitive advantage. The positive impact of social workers with well-adjusted competences will be noticeable in a well-performed service at a professional level, maintaining all required standards of social services. The usability of the proposed schemes was tested for its validation, demonstrating practical benefits for the management of continuing education in social services.

Systematic continuing education is an important element of human resource management. It allows us to evaluate the cycle of continuing education and streamline its individual parts. Research results pointed to other facts that influence the systematic process of competence extension, which were implemented in the diagram shown in Figure 1. Continuing education in social services is linked to strategic education plans, staff development plans, motivations plans and a set of required competences for a social services job. At the same time, as the diagram shows, it is necessary to reflect these specifics, especially the development plans, the organization’s strategic training plans, and the required competences, in evaluating continuing education.

This research introduced the expansion of competences in the social services field and proposed schemes for managing the expansion of social workers’ competences. Competences are key to the social work profession. Competences are not acquired forever; a worker can gain, develop, or lose them again if they do not consolidate them. Professional competences play a key role in social work. Social work requires a comprehensive professional and social preparation of the social worker. Social competences ensure, to some extent, how professional competences will be applied in practice. Therefore, it is a comprehensive complex, which is why all professional competences were examined in the research. Social competence should be the focus of further research. The research has some limitations. The data collected represent a sample of social service workers in the Czech Republic. As this is a professionally oriented group, these results can contribute to the management of the expansion of competences in the field of social services in the Czech Republic. Another area for improvement is the size of the research sample. Although all organizations were contacted according to the Register of Social Service Providers in the Czech Republic, the return rate was 21%. Future research should build on our research and focus on expanding the research sample.

## Data availability statement

The datasets analysed for this research can be found at Zenodo: https://zenodo.org/record/7863867.

## Ethics statement

Ethical review and approval was not required for the research on human participants in accordance with the local legislation and institutional requirements. Written informed consent for participation was not required for this research in accordance with the national legislation and the institutional requirements.

## Author contributions

GJP contributed to the conception and design of the research, methodology, model creation, organized the database, and performed the statistical analysis. GJP and KZ prepared and wrote the first draft of the manuscript, wrote sections of the manuscript. All authors contributed to the article and approved the submitted version.

## Conflict of interest

The authors declare that the research was conducted in the absence of any commercial or financial relationships that could be construed as a potential conflict of interest.

## Publisher’s note

All claims expressed in this article are solely those of the authors and do not necessarily represent those of their affiliated organizations, or those of the publisher, the editors and the reviewers. Any product that may be evaluated in this article, or claim that may be made by its manufacturer, is not guaranteed or endorsed by the publisher.
